# Broad-spectrum rescue compounds for structural p53 mutations: *perspective on ‘Arsenic trioxide rescues structural p53 mutations through a cryptic allosteric site*’

**DOI:** 10.1093/jmcb/mjab007

**Published:** 2021-01-28

**Authors:** Jia-Le Wu, Shuo Chen, Min Lu

**Affiliations:** Shanghai Institute of Hematology, State Key Laboratory of Medical Genomics, National Research Center for Translational Medicine (Shanghai), Ruijin Hospital affiliated to Shanghai Jiao Tong University School of Medicine, Shanghai 200025, China

Precision medicine targeting gene mutations holds the promise of changing the landscape of cancer care and prognosis, but currently approved drugs in this category are efficacious in only a very small percentage of all cancer patients (Tannock and Hickman, 2016). *TP53*, encoding the tumor suppressor and transcription factor p53, is the most frequently mutated gene in human cancers (Joerger and Fersht, 2016; Sabapathy and Lane, 2018; Levine, 2019). Pharmacologically rescuing mutant p53 by restoring wild-type function could therefore potentially be widely applicable in cancer treatment and is considered to be a holy grail of cancer research (Joerger and Fersht, 2010). Indeed, at least 17 compounds that can rescue mutant p53 variants were reported by 2018 (Sabapathy and Lane, 2018). Unfortunately, p53 mutations still remain therapeutically nonactionable due to challenges such as heterogeneous mechanisms of inactivation by different mutations and the absence of obvious targetable drug-binding pockets (except Y220C mutant). In a recent publication (Chen et al., 2021), we reported the identification of small-molecule compounds that rescue a broad class of p53 mutations. Notably, these include arsenic trioxide (ATO), which is used to treat acute promyelocytic leukemia (de Thé et al., 2017). The study differentiates itself from previous reports in: (i) rescuing mutant p53 at striking levels when benchmarked against previously reported rescue compounds; (ii) providing a structural mechanism, wherein the arsenic atom binds to a cryptic allosteric site connecting the loop–sheet–helix (LSH) motif with the β-sandwich skeleton to increase the thermostability of mutant p53; (iii) offering a largely defined spectrum of applicable p53 mutations—the structural mutations that compromise the wild-type structure of p53 and collectively account for more than half of all clinically relevant p53 alterations.

In this essay, we further focus on the broad-spectrum rescue compounds for structural p53 mutants, rather than ATO itself. We will discuss our opinions on future prospects in drug discovery and the challenges faced by both basic and translational research on this type of p53-rescuing compounds. The Y220C-targeting compounds that specifically rescue the individual Y220C mutation and the DNA contacting mutation-rescuing compounds that remains elusive for the rescuing mechanism at the atomic level are not discussed here.

At the drug discovery stage, we normally apply three easily accessible and highly sensitive assays to validate a hit achieved in library screens—the differential scanning fluorimetry assay, PAb1620 immunoprecipitation assay, and luciferase reporter assay—which are respectively used to assess the thermostability, folding status, and transcriptional activity of p53. However, we noticed that mutants such as V272M, R282W, and E285K are relatively inert in PAb1620 epitope promotion despite substantial increases of thermostability, while mutants such as R175H, R249S, and H179Y are relatively inert in terms of transcriptional activity promotion. After validation, we normally rely on three go-to criteria to decide whether to push the rescue compounds into animal and preclinical studies—the availability of a co-crystal structure, a structure‒activity relationship consistent with crystal structure, and target specificity in cells. We frequently encounter compounds that generate reproducible positive readouts in rescuing some aspects of p53 activity in the library screens, but <1% of the compounds meet all three criteria, particularly the criterion of target specificity. For example, compounds preferentially inhibiting isogenic cell lines with mutant p53 are frequently found to actually target p53 mutation-associated downstream or compensatory events, rather than the mutant p53 itself. Similarly, compounds able to increase the thermostability of mutant p53 *in vitro* are frequently found to have off-target activity and thus are unable to bind to the desired target with sufficient amount inside living cells. Similar dilemmas were encountered when we obtained rescue compounds for mutations of *PTEN*, *ATM*, *RB1*, or *CDKN2A* in the library screens.

A major challenge faced by basic research lies in obtaining a detailed understanding of the DNA-binding surface of the pharmacologically rescued mutant p53 at the atomic level (Figure 1A). A puzzling observation in our ATO study is that many structural p53 mutants, such as R249S, restore only limited transcriptional activity despite successful global refolding by ATO (Chen et al., 2021). Co-crystal structures revealed that the arsenic-bound R249S DNA-binding domain has a distorted DNA-binding L3 loop. Since DNA binding is the prerequisite for p53 to perform its transcriptionally regulatory function, dissecting the DNA-binding surface of rescued p53 mutants at the atomic level may help explain the puzzling observations. This may also help explain the difference of transcriptional target selectivity (if present) between wild-type p53 and rescued mutant p53. We believe that obtaining crystal structures of pharmacologically rescued mutant p53 variants in complex with the DNA can provide the ultimate answer, although it is challenging and so far, there is no such p53 structure deposited in the PDB database (http://www.wwpdb.org/). Obtaining a crystal structure of the cpd/p53/DNA triple complex may have further implications for the development of drugs targeting transcription factors, most of which are still undruggable (except nuclear receptors) (Bushweller, 2019). So far, directly modulating the DNA-binding activity of transcription factors using small-molecule compounds still remains highly challenging (Bushweller, 2019).

**Figure 1:**
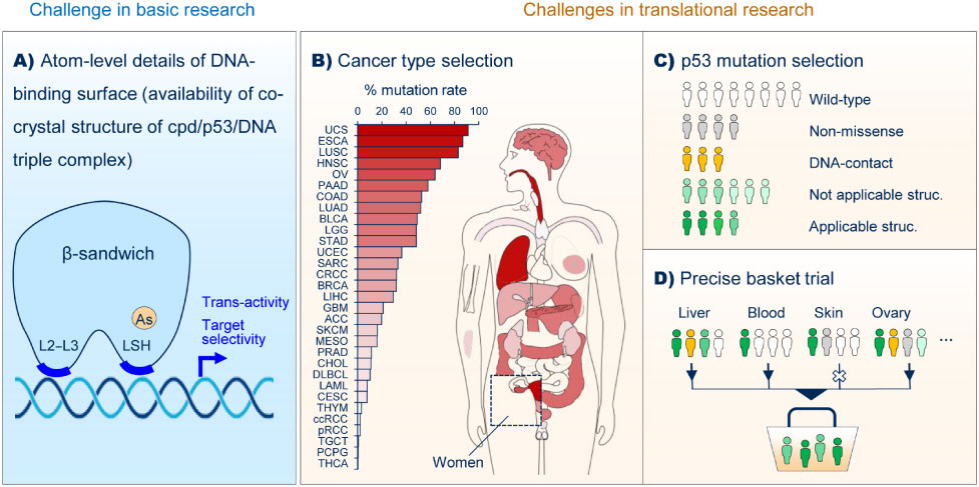
Challenges in the development of broad-spectrum rescue drugs for structural p53 mutants in basic and translational research. (**A**) Understanding the details of the DNA-binding surface of the pharmacologically recued p53 mutants at the atomic level. (**B** and **C**) Selecting appropriate cancer types for clinical trials based on their dependency on the p53 mutation for growth and survival, together with the applicable p53 mutations based on experimental evidence. In **B**, the organs are colored according to p53 mutation frequency of each cancer type in the TCGA Pan-Cancer Altas Studies. (**D**) Proposed structure of a precise basket trial for the broad-spectrum rescue drugs for mutant p53.

Two major challenges faced in the related translational research are the selection of cancer types and mutations for clinical trials (Figure 1B and C). This is mainly caused by the wide-spectrum cancer distribution of p53 mutations, which show a veritable ‘rainbow’ of features regarding the rescue effectiveness and efficiency by the same rescue compound (Sabapathy and Lane, 2018). These challenges apparently do not exist for the widely used epidermal growth factor receptor inhibitors and the recent ground-breaking KRAS-G12C inhibitors (Canon et al., 2019) since, according to The Cancer Genome Atlas Program Pan-Cancer Altas Studies, their applicable mutations are predominately found in one cancer type (lung adenocarcinoma) and clustered in one or a few codons of their encoding genes. Under ideal circumstances, the trials should recruit patients with a type of cancer highly dependent on p53 mutations; in other words, the p53 mutation should be a key driver in this cancer type and the cancer cells depend on the p53 mutation to survive and/or grow. Previous clinical trials have confirmed the importance of selecting appropriate cancer types for a targeted drug. For example, BRAFV600E inhibitors exhibit high efficacy in treating BRAF-mutant melanoma but not colorectal cancers (Kopetz et al., 2015). How to predict or identify the cancer types sensitive to mutant p53-rescuing drugs? Are these cancer types those having a high p53 mutation frequency such as ovarian cancer, those having a high hazard ratio such as hematological malignancies, those having a higher p53 mutation frequency upon relapse, or the frequent spontaneous cancer types in p53-defective mice (lymphoma) and Li-Fraumeni Syndrome people (sarcoma)? The next challenge is to select the p53 mutations that can be effectively and, ideally, highly efficiently rescued by the drug. Our study suggests that ATO specifically rescues structural mutations, but not all structural mutations can be rescued. There is an apparent trend (unpublished data) that ATO is most effective on large-to-small amino acid mutations, mutations near the arsenic-binding pocket, temperature-sensitive mutations, as well as the mutations occurring on hydrophobic residues and in the LSH motif. Thus, experimentally determining the list of ATO-applicable targets among thousands of p53 mutations is a challenge that should be taken in the near future. We thus propose an ideal trial mode upon addressing these two challenges—a precise basket trial for numerous cancer types selectively recruiting patients with sensitive cancer types and ATO-applicable p53 mutations (Figure 1D).

*[We thank the staff of the BL17U/BL18U1/BL19U1/BL19U2/BL01B beamlines of National Center for Protein Science Shanghai (NCPSS) at the Shanghai Synchrotron Radiation Facility (SSRF). M.L. was funded by the National Key R&D Program of China (2017YFA0506200), the National Natural Science Foundation of China (82073292, 81622002, and 81861130368), the Gaofeng Clinical Medicine Grant (828318), the Shanghai Excellent Youth Academic Leader (20XD1422700), the Shanghai Medical and Health Excellent Discipline Leader Development Plan (2018BR36), the Shanghai Collaborative Innovation Center for Translational Medicine (TM201902), the Foundation of the National Facility for Translational Medicine (Shanghai) (TMSK-2020-003), and the Newton Advanced Fellowship (NAF\R1\180216). J.-L.W., S.C., and M.L. wrote the manuscript.]*

## References

[mjab007-B1] Bushweller J.H. (2019). Targeting transcription factors in cancer—from undruggable to reality. Nat. Rev. Cancer 19, 611–624. 31511663 10.1038/s41568-019-0196-7 PMC8820243

[mjab007-B2] Canon J. , Rex K., Saiki A.Y., et al (2019). The clinical KRAS(G12C) inhibitor AMG 510 drives anti-tumour immunity. Nature 575, 217–223. 31666701 10.1038/s41586-019-1694-1

[mjab007-B3] Chen S. , Wu J.-L., Liang Y., et al (2021). Arsenic trioxide rescues structural p53 mutations through a cryptic allosteric site. Cancer Cell *39*, 225–239.e8. 10.1016/j.ccell.2020.11.013 33357454

[mjab007-B4] de Thé H. , Pandolfi P.P., Chen Z. (2017). Acute promyelocytic leukemia: a paradigm for oncoprotein-targeted cure. Cancer Cell 32, 552–560. 29136503 10.1016/j.ccell.2017.10.002

[mjab007-B5] Joerger A.C. , Fersht A.R. (2010). The tumor suppressor p53: from structures to drug discovery. Cold Spring Harb. Perspect. Biol. 2, a000919. 20516128 10.1101/cshperspect.a000919 PMC2869527

[mjab007-B6] Joerger A.C. , Fersht A.R. (2016). The p53 pathway: origins, inactivation in cancer, and emerging therapeutic approaches. Ann. Rev. Biochem. 85, 375–404. 27145840 10.1146/annurev-biochem-060815-014710

[mjab007-B7] Kopetz S. , Desai J., Chan E., et al (2015). Phase II pilot study of vemurafenib in patients with metastatic BRAF-mutated colorectal cancer. J. Clin. Oncol. 33, 4032–4038. 26460303 10.1200/JCO.2015.63.2497 PMC4669589

[mjab007-B8] Levine A.J. (2019). Targeting therapies for the p53 protein in cancer treatments. Ann. Rev. Cancer Biol. 3, 21–34.

[mjab007-B9] Sabapathy K. , Lane D.P. (2018). Therapeutic targeting of p53: all mutants are equal, but some mutants are more equal than others. Nat. Rev. Clin. Oncol. 15, 13–30. 28948977 10.1038/nrclinonc.2017.151

[mjab007-B10] Tannock I.F. , Hickman J.A. (2016). Limits to personalized cancer medicine. N. Engl. J. Med. 375, 1289–1294. 27682039 10.1056/NEJMsb1607705

